# Supplementary material for: TLR5 Variants Are Associated with the Risk for COPD and NSCLC Development, Better Overall Survival of the NSCLC Patients and Increased Chemosensitivity in the H1299 Cell Line
Source: Biomedicines. 2022 Sep 9;10(9):2240. doi: 10.3390/biomedicines10092240 (PMC9496592; doi:10.3390/biomedicines10092240)
Supplement: Supplementary file 1 [file biomedicines-10-02240-s001.zip › Supplementary Tables S2 and S3.pdf]

Supplementary Table S2. List of statistically significant differentially expressed genes in H1299 and WI-38 cell lines. Differential expression was calculated by DESeq2 package (R program) using raw data obtained by the sequencing of RNA isolated from the cells overexpressing either $TLR5^{WT}$ or $TLR5^{N592S}$ gene variant.

Cell line/Status	Gene Symbol	Gene function[1]	log2FC	p_{adj}[2]
Up-H1299	ZBTB12	- predicted to be involved in regulation of transcription by RNA polymerase II; known as sequence-specific silencers of gene expression.	8.655886	8.76E-08
	C1QTNF3	- involved in cellular triglyceride homeostasis, negative regulation of NF-kappaB signaling and regulation of cytokine production.	7.789553	0.000809
	GPSM3	- involved in positive regulation of inflammatory response; predicted to act within positive regulation of cytokine production involved in inflammatory response and positive regulation of leukocyte chemotaxis.	5.427362	0.031135
Dow-H1299	MAST4	- member of the microtubule-associated serine/threonine protein kinases; contain a domain that gives the kinase the ability to determine its own scaffold to control the effects of their kinase activities	-2.37413	3.23E-05
	SLC19A3	- ubiquitously expressed thiamine transporter associated with biotin-responsive basal ganglia disease (BBGD), a recessive disorder manifested in childhood that progresses to chronic encephalopathy, dystonia and quadriparesis.	-2.79038	0.031135
	AC025048	- involved in protein deubiquitination.	-6.45133	0.019739
Up-WI-38	H2AC19	- member of the histone H2A family; histones play a central role in transcription regulation, DNA repair, DNA replication and chromosomal stability	3.376579	1.85E-06
	PKIA	- member of the cAMP-dependent protein kinase (PKA) inhibitor family; protein interact with and inhibit the activities of both C-alpha and C-beta catalytic subunits of the PKA.	2.845436	0.000134
	CHRFAM7A	- expressed in the neuroendocrine cells in the lung; the role of the proteins seems to be as a modulator of inflammatory responses, where its activation prevents release of cytokines such as TNFα, IL-6, IL-8 and high mobility group B protein-1 (HMGB1)	2.796132	0.027919
	GPER1	- member of the G-protein coupled receptor 1 family; receptor binds estrogen and activates multiple downstream signaling pathways, leading to stimulation of adenylate cyclase and an increase in cyclic AMP levels; receptor has been shown to play a role in diverse biological processes, including bone and nervous system development, metabolism, cognition, male fertility and uterine function.	2.787058	0.036375
	ARHGAP4	- member of the rhoGAP family of proteins which play a role in the regulation of small GTP-binding proteins belonging to the RAS superfamily; genomic deletions of this gene have been found in patients with nephrogenic diabetes insipidus.	2.723753	0.049163
	LIME1	- transmembrane adaptor protein that links the T and B-cell receptor stimulation to downstream signaling pathways via its association with the Src family kinases Lck and Lyn, respectively.	2.617301	0.003832
	DMBT1	- loss of transcript is detected in medulloblastoma cell line and associated with the progression of human cancers; different gene transcripts are expressed in fetal and adult lung; protein precursor is a glycoprotein containing multiple scavenger receptor cysteine-rich (SRCR) domains; transcript variant of 8.0	1.854898	0.011504

		kb has been shown to bind surfactant protein D; predicted to play a role in the interaction of tumor cells and the immune system.		
	PRR7	- Protein tyrosine kinase binding activity and ubiquitin-like protein ligase binding activity; involved in negative regulation of protein ubiquitination and positive regulation of apoptotic process.	1.83814	0.016745
	RAB43	- involved in Golgi organization and phagosome maturation..	1.801713	0.038722
	MIF	- encodes a lymphokine involved in cell-mediated immunity, immunoregulation and inflammation; plays a role in the regulation of macrophage function in host defense through the suppression of anti-inflammatory effects of glucocorticoids; form the complex with the JAB1 protein in the cytosol near the peripheral plasma membrane, indicateing the role in integrin signaling pathways.	1.735825	0.040099
	ZNF287	- encodes a member of the krueppel family of zinc finger proteins, suggesting a role as a transcription factor; ubiquitously expressed in the lungs.	1.730281	0.000442
	TCF19	- encodes a protein that contains a PHD-type zinc finger domain and likely functions as a transcription factor; encoded protein plays a role in the cell proliferation and apoptosis.	1.694099	0.01666
	HMCN1	- encodes a large extracellular member of the immunoglobulin superfamily and its thought to play in many cellular processes such as stabilization of the germline syncytium, anchorage of mechanosensory neurons to the epidermis, and organization of hemidesmosomes in the epidermis.	1.679007	0.004647
	MEGF6	- encodes an oncogene which very likely promote cell proliferation and inhibit apoptosis; encoded protein also accelerate cell migration via TGFβ/SMAD signaling-mediated EMT.	1.675893	0.036481
	CHST12	- encoded by gene belongs to the sulfotransferase 2 family; catalyzes the transfer of sulfate to position 4 of the N-acetylgalactosamine (GalNAc) residue of chondroitin and desulfated dermatan sulfate.	1.651354	0.041875
	FBLN2	- encodes an extracellular matrix protein, which belongs to the fibulin family; may play a role during organ development, in particular, during the differentiation of heart, skeletal and neuronal structures.	1.577472	0.027297
	HIC1	- functions as a growth regulatory and tumor repressor gene; hypermethylation or deletion of the region of this gene have been associated with tumors and the contiguous-gene syndrome.	1.574313	0.007652
	PAM16	- encodes a mitochondrial protein involved in granulocyte-macrophage colony-stimulating factor (GM-CSF) signaling; regulate the import of nuclear-encoded mitochondrial proteins into the mitochondrial matrix and may be important in reactive oxygen species (ROS) homeostasis.	1.537559	0.002418
**Down-WI-38**	ZNF493	- enable DNA-binding transcription activator activity and predicted to be involved in regulation of transcription by RNA polymerase II; ubiquitous expression in the lungs.	-1.93133	0.032311
	ZNF391	- enable DNA-binding transcription activator activity and predicted to be involved in regulation of transcription by RNA polymerase II; ubiquitous expression in the lungs.	-2.01123	0.008988
	OBSCN	- encodes a protein that belongs to the family of giant sacromeric signaling proteins including titin and nebulin, and may have a role in the organization of	-2.49535	0.005849

	myofibrils during assembly and may mediate interactions between the sarcoplasmic reticulum and myofibrils.		
RNF32	- encodes protein that contains two RING finger motifs present in a variety of functionally distinct proteins and are known to be involved in protein-DNA or protein-protein interactions.	-3.02556	0.017417
PBX2	- encodes a ubiquitously expressed member of the TALE/PBX homeobox family; protein is a transcriptional activator which binds to the TLX1 promoter.	-3.26	0.000203
INSR	- encodes a member of the receptor tyrosine kinase family of proteins; binding of insulin or other ligands to receptor activates the insulin signaling pathway, which regulates glucose uptake and release, as well as the synthesis and storage of carbohydrates, lipids and protein.	-3.57901	0.001722
LAMP3	- encodes lysosomal membrane glycoprotein which contributes to protein degradation and cell survival during proteasomal dysfunction; modulatie the autophagic process, promotes hepatocellular lipogenesis through activation of the PI3K/Akt pathway, play a role in dendritic cell function and in adaptive immunity, plays a positive role in post-entry steps of virus replication, either virus uncoating, cytosolic transport, or nuclear import of viral components.	-3.83812	0.042104

[1] - short functional relevance of identified genes taken from the available databases (www.genecards.org; www.uniprot.org; www.ncbi.nlm.nih.gov; www.pubmed.ncbi.nlm.nih.gov)

[2] - p value adjusted for multiple testings using Benjamini-Hochberg method

Supplementary Table S3. Gene set enrichment analysis for KEGG pathways in H1299 and WI-38 cell line indicating the effect of N592S variant on pathway enrichment

Name	FDR q-value	Enrichment score	Description
H1299_long term depression	0.017078	-1.5182894	Process involving a decrease in the synaptic strength between parallel fiber (PF) and Purkinje cells (PCs). Multiple signal transduction pathways have been shown to be involved in this process triggering a cascade of events culminating in a phosphorylation-dependent, long-term reduction in AMPA receptor sensitivity at the PF-PC synapse.
H1299_antigen processing and presentation	0.025194	-1.4960678	Activation of MHC I and MHC II molecules that activate CD8 and CD4 T cells and NK cells.
H1299_aldosterone regulated sodium reabsorption	0.038095	-1.448107	Aldosterone, a steroid hormone, regulates renal Na+ reabsorption and, therefore, plays an important role in the maintenance of electrolyte and fluid homeostasis
H1299_natural killer cell mediated cytotoxicity	0.042227	-1.3609923	Natural killer (NK) cells are part of the innate immune system and are involved in early defenses against both allogeneic cells and autologous cells undergoing various forms of stress, such as infection with viruses, bacteria, or parasites or malignant transformation.
H1299_ABC transporters	0.042553	-1.4712279	ATP-binding cassette (ABC) transporters that couple ATP hydrolysis to active transport of a wide variety of substrates such as ions, sugars, lipids, sterols, peptides, proteins, and drugs.
WI-38_calcium signaling pathway	0.043796	-1.3438206	Calcium ion (Ca2+) participates in regulating some important physiological activities in the human body, including the nervous system excitability, the contraction of muscles, the intestinal microbial activity and the activity of enzymes. Ca2+ can act in signal transduction resulting from activation of ion channels or as a second messenger caused by indirect signal transduction pathways such as G protein-coupled receptors.
WI-38_tyrosine metabolism	0.035587	1.5060816	Tyrosine is an amino acid important in the synthesis of thyroid hormones, catecholamines, and melanin.
